# An Orthopedic Healthcare Facility Governance Assessed with a New Indicator System

**DOI:** 10.3390/healthcare12111080

**Published:** 2024-05-24

**Authors:** Flaviu Moldovan, Liviu Moldovan

**Affiliations:** 1 Orthopedics—Traumatology Department, Faculty of Medicine, “George Emil Palade” University of Medicine, Pharmacy, Science, and Technology of Targu Mures, 540142 Targu Mures, Romania; 2 Faculty of Engineering and Information Technology, “George Emil Palade” University of Medicine, Pharmacy, Science, and Technology of Targu Mures, 540142 Targu Mures, Romania; liviu.moldovan@umfst.ro

**Keywords:** healthcare facility governance, sustainability, orthopedics, reference framework, healthcare facility, assessment

## Abstract

Background and Objectives: A sustainability-oriented hospital governance has the potential to increase the efficiency of healthcare services and reduce the volume of expenses. The objective of this research is to develop a new complex tool for evaluating healthcare facility governance as a component of social responsibility, integrated into sustainability. Materials and Methods: We designed the research to develop the domains of a new reference framework for evaluating healthcare facility governance. The methodology for designing the indicators that make up the new reference framework consists of collecting and processing the most recent and relevant practices regarding the governance of healthcare facilities that have been reported by representative hospitals around the world. Results: We designed eight indicators that are brought together in the healthcare facility governance indicators matrix. They have descriptions and qualitative and quantitative rating scales with values from 0 to 5 that allow the degree of fulfillment to be quantified. The importance of the indicators is evaluated on a specific scale described qualitatively and quantitatively by values from 0 to 5. The values of the degree of achievement–importance couples of the indicators allow the development of improvement measures with priority according to the results revealed by the Eisenhower-type assessment diagram. Conclusions: Validation in practice of the system of indicators at an emergency hospital in an orthopedic profile highlighted the fact that they can be integrated into other national and international reference frameworks implemented in the hospital. The added value of the implementation consists of the facilitation of sustainable development and the orientation of health personnel, patients, and interested parties toward sustainability.

## 1. Introduction

To sustain the market share of nonprofit hospitals, regulators, managers, administrators, and physicians must pay attention to the governance function of hospitals and the structures through which it is achieved. In general, the organizational structures of hospitals are preserved for longer periods, while the mode of financing and technological developments change continuously. Accountability for hospital performance requires increased board control over managers and physicians. But to accelerate public hospital reform the Healthcare Commission needs more resources, tools, and political support, which contribute to equitable access to quality care at affordable prices [1]. The use of appropriate hospital governance strategies has the potential to support increasing the efficiency of healthcare services and reducing the length of time patients spend in hospitals [2].

The German, British, and French healthcare systems underwent several reforms after the 1980s that were oriented toward their commercialization. The objective was to change the governance of hospitals with the support of “merchant” reforms that, with the insertion of market logic, have the potential to increase the efficiency of existing expenditures and reduce the need for public expenditures [3,4]. But overall reforms have addressed the challenges facing the system and generated modest results. The remaining shortcomings of the healthcare system highlighted the limitations of the evolutionary approach to reforms and suggested that a more revolutionary approach is needed that starts from the bottom up by resizing hospital governance [5]. From this perspective, it is necessary to analyze the efficiency in healthcare expenses of innovative hospital management models such as Public Private Partnerships or Private Finance Initiatives with the support of new tools dedicated to hospital governance models [6]. They could be more efficient in healthcare spending and would be a useful tool in planning new healthcare infrastructures.

But there is a need to change governance models by empowering and involving patients in the management of healthcare facilities. In this way, their joint responsibility in the development of healthcare services is created. They should evolve from the traditional view of paternalistic medicine toward a more participatory and informed patient–provider relationship. New models for promoting patient-centered methods, efficiency, effectiveness, ensuring system sustainability, and a high level of patient satisfaction require excellence in three main areas of improvement: clinical knowledge, methodology, and patient experience [7]. Facilitating hospital governance can be supported by investigating the technical efficiency of hospital care using dedicated methods such as Data Envelopment Analysis [8]. However, the relationship between access to healthcare, quality, and efficiency is far from being well defined. In the literature, there is no consensus regarding the existence of a trade-off between hospital performance and its social dimensions, which include access to medical care, the appropriate nature of care, and the safety of the medical act. For this, a new dimensioning of governance is needed that also includes this responsibility [9].

From this perspective, an incentive is needed to promote quality improvement processes in hospitals and in the community with the support of new institutionally accredited governance models [10]. There is a need for new evaluation tools and new reference frames, which, with the support of indicators and digital applications, can achieve this desired change [11]. The new governance models applied in hospitals that meet the requirements of the patient-centered care process and have a high potential for improvement in providing medical care are demonstrated including by lower mortality rates in orthopedic departments [12].

With the support of these controversies presented by the scientific literature regarding healthcare facility governance, in our study, we formulated the following research questions:

(RQ1): What are the aspects that define the sustainability of healthcare facilities governance?

(RQ2): What are the healthcare practices validated in globally representative hospitals regarding the governance of healthcare facilities?

(RQ3): What indicators can be defined to evaluate the healthcare facility governance?

(RQ4): How should indicators be defined to assess and monitor the progress of healthcare facility governance?

With the support of these research questions, we developed the main objective of this research. It consists of the development of a new complex tool for evaluating the healthcare facility governance, as a component of social responsibility, integrated into sustainability.

A secondary objective of the research is to ensure the compatibility of the newly developed evaluation instrument with the national accreditation legislation but also with other reference frameworks currently used in hospitals.

## 2. Materials and Methods

**Figure 1:**
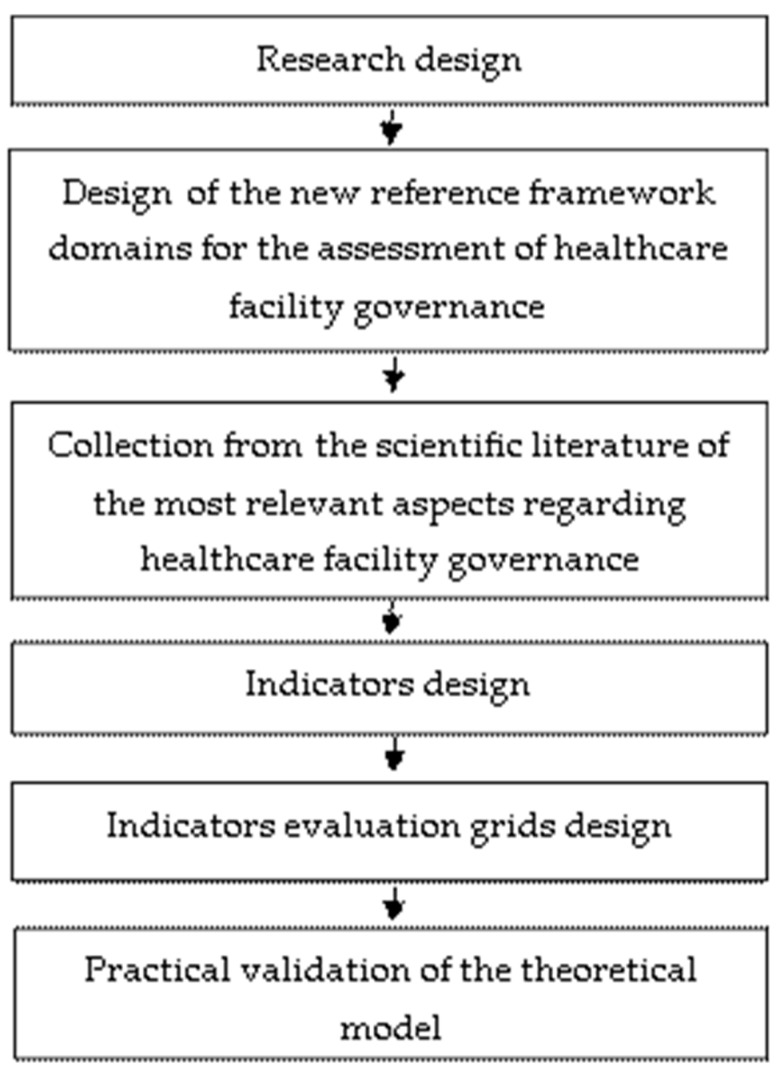
Flowchart with the steps of the research methodology.

The steps of the research methodology we have used are presented in Figure 1.

### 2.1. The Research Design

We designed this qualitative and primary exploratory research to investigate the research questions formulated in the introductory section of our study. We collected the most recent and relevant issues related to healthcare facility governance that were reported by representative hospitals from all over the world from the scientific literature. The collection of specific research data and their validation in the practice at an orthopedic hospital was achieved by following the guidelines of the Declaration of Helsinki. The conduct of the study was approved by the Ethics Committee of the Emergency County Hospital Targu Mures (ECHTM).

### 2.2. The Reference Framework Areas

Healthcare facility governance is made up of a multitude of specific aspects that have been highlighted through the study of the specialized medical literature and presented in the introductory section of this research. Their adequate treatment, in order to improve the quality of medical services, requires a new appropriate reference framework, which, through performance indicators, allows for the periodic evaluation of hospitals. In this way, based on the audit reports, improvement programs can be drawn up. 

Currently, there are national legal norms for the accreditation of services in healthcare facilities with beds [13] but also for the accreditation of outpatient healthcare services [14]. At the international level, global models developed through international partnerships are intended to evaluate the quality of medical services in hospitals [15], without addressing sustainability requirements.

Having this requirement as a starting point, during the research, we established the domains of a new Health–Sustainability (H–S) reference framework, so that it is compatible with the international reference frameworks [15] but also the national ones [13,14], which are currently applied in hospitals.

**Figure 2:**
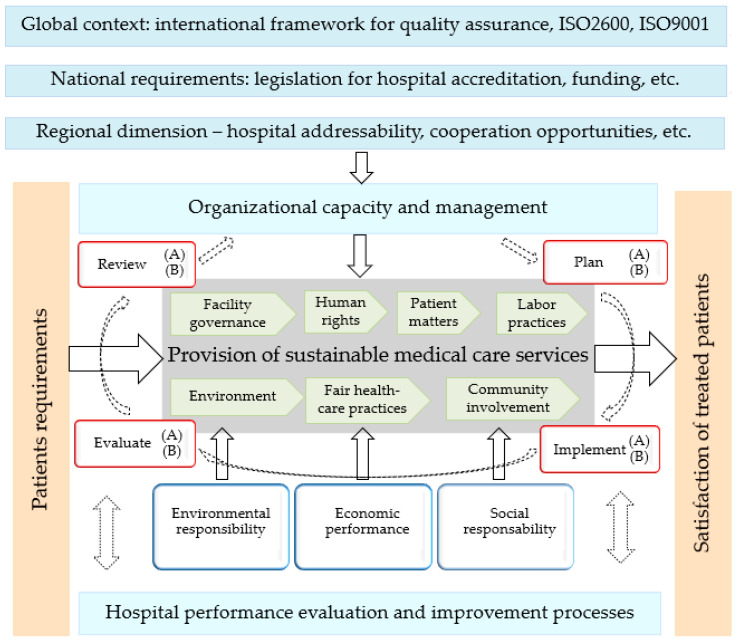
The domains of the reference framework Health–Sustainability (H–S) and the quality cycle of basic medical activities regarding healthcare facility governance: Plan: (A) Hospital institutional accreditation, (B) Designing patient-oriented medical services; Implement: (A) Provision of medical services, (B) Patient transfer provision; Evaluation: (A) Local opinion leaders’ evaluation and involvement, (B) Satisfaction assessment for patients and staff; Review: (A) Staff self-assessment, (B) Medical services innovation.

In this sense, we considered the three classic areas of sustainability: environmental, economic, and social. The innovative aspect proposed in this research deals with social aspects in accordance with the seven requirements of the social responsibility standard ISO 26000 [16]. Social aspects were adapted to the basic processes of the hospital regarding the provision of sustainable healthcare services, as follows, healthcare facility governance, human rights [17], labor practices [18], environment [19], fair healthcare practices [20], patient matters [21], and community involvement and development (Figure 2).

Corresponding to the content of medical activities, in our model, we ordered the 4 stages of the quality cycle in accordance with the requirements of the ISO9001 quality assurance standard [22]. In each stage of the quality cycle, we designed two basic medical activities. Thus, in the planning phase, the basic medical activities are (A) hospital institutional accreditation and (B) designing patient-oriented medical services. These are followed in the implementation phase by the activities (A) provision of medical services and (B) patient transfer provision. The third evaluation phase consists of the activities: (A) local opinion leaders’ evaluation and involvement and (B) satisfaction assessment for patients and staff. The final review phase consists of (A) staff self-assessment and (B) medical services innovation.

### 2.3. Data Collection and Analysis

In this study, we collected the necessary data from the representative medical and managerial scientific literature describing activities related to healthcare facilities’ organizational governance. It followed the Preferred Reporting Items for Systematic Reviews and Meta-Analyses (PRISMA) guidelines. We searched the following databases for eligible studies: PubMed, Web of Science, and EMBASE (OVID). Searches through January 2024 were conducted with no publication date or language restrictions. The following keywords were applied to the search: (hospital governance or accreditation or governance strategy) AND (hospital management or healthcare commission or board of directors) AND (health reform or hospital performance or health expenses). 

**Figure 3:**
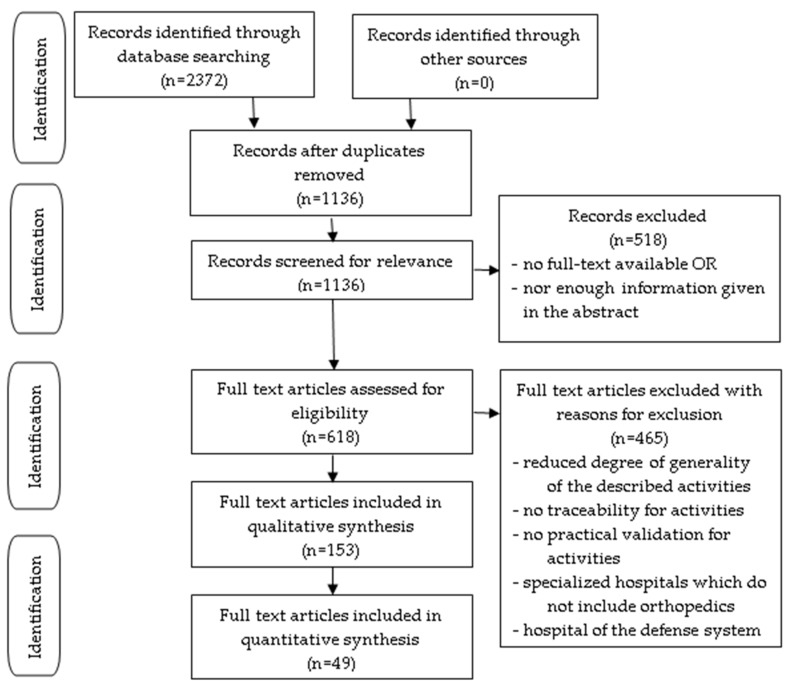
PRISMA Flowchart for the systematic review detailing the number of records screened and the full texts retrieved.

For the qualitative analysis, we included studies describing activities validated in practice with a high degree of generality that can be applied in different healthcare facilities and that allow traceability. No publication date, language, or publication status restrictions were imposed. Each study was evaluated for inclusion in the quality cycle stages. The outcome results were the activities for governance design, governance provision, governance evaluation, and governance continuous improvement. The two authors screened the titles and abstracts of all references independently. Potentially relevant articles were retrieved with the support of an eligibility checklist for inclusion in the analysis. Any disagreements were resolved by consensus. The reasons for exclusion were included in a PRISMA flowchart (Figure 3). An eligibility checklist was used for the inclusion of activities in the stages of the quality cycle.

In this way, we created a database with the most relevant aspects of healthcare facility governance. This was processed with Microsoft Office support. Then, we ordered and analyzed distinctly the 8 basic medical activities from the quality cycle Plan (A-B), Implement (A-B), Evaluate (A-B), and Review (A-B).

### 2.4. Validated Evidence for Healthcare Facility Governance

In the following sections, the representative medical practices related to healthcare facility governance that were collected from the scientific literature and analyzed to design the indicators of the new frame of reference are presented in the sequence of the quality cycle stages.

#### 2.4.1. Indicators for Healthcare Facility Governance Design

A prerequisite for the scientific management of hospitals is accreditation [23]. This facilitates the achievement of quality and patient safety objectives, due to compliance with the standards that guide excellence in public healthcare services [24]. Shakibaei [25] shows that the hospital accreditation process contributes to positive changes in hospital process management cycles. However, the successful implementation of the accreditation program requires a detailed assessment of the financial and technical needs of the hospitals, as well as the development of a plan to meet these needs. Recently, there has been recognition of the value of accreditation also in low- and middle-income countries due to promotion by the World Health Organization. But with the rapid pace of change in society, it must be reformed and repositioned, with the support of decision-making structures and processes [26]. 

Mosadeghrad and Ghazanfari [27] developed a comprehensive model for hospital accreditation that contains ten constructs. These are categorized into seven constructs that include hospital management and leadership as well as another three, which are hospital, employee, and patient outcomes. The accreditation model requires adequate attention to systems thinking but also to structures, processes, and outcomes. The stages of integration of hospital accreditation requirements are sequential and interconnected. These are influenced by management involvement, teamwork, and organizational culture. At the same time, it is necessary to understand the accreditation sequences and the mechanisms by which accreditation requirements are routinized in operations [28].

With the support of these healthcare practices, we developed the content of the indicator PA1–decision-making structures and processes. Its contents are shown in Table A1. This indicator is used to evaluate the basic medical activity of the hospital, located in the Plan (A) stage.

Edgman-Levitan et al. [29] show that designing healthcare quality assurance processes requires actively understanding what patients value. For this, there are a number of methods of gathering patient requirements that need to be widely applied and patients need to be treated as full partners in their care. The patient may be satisfied with the hospital environment due to the healing environment and due to the human interactions [30]. Hospital design requires combining the patient’s perceptions and expectations of the physical environment in the care area. Space and architecture influence the emotions of the patient and his relatives, as well as the providers of medical services [31].

Patient-centered care innovation in orthopedics requires the application of design thinking at all stages of orthopedic innovation development, from exploring specific challenges to introducing new technological breakthroughs in the musculoskeletal health market [32]. Patient-centered care can be supported by telemedicine but this requires greater specificity [33]. The burden of musculoskeletal pain can be alleviated through patient education and healthcare literacy [34]. 

With the support of these healthcare practices, we developed the content of the indicator PB1–Healthcare quality assurance processes design. Its contents are shown in Table A3. This indicator is used to evaluate the basic medical activity of the hospital, located in the Plan (B) stage.

#### 2.4.2. Indicators for Healthcare Facility Governance Provision

The impetus for the use of the electronic healthcare record came as a result of some legislative initiatives regarding information technology in healthcare. It provides evidence-based clinical decision support to facilitate patient care. A series of tools are used, such as computerized structured recording of doctor’s recommendations, order sets, alerts, templates, and reminders [35]. El-Dawlatly et al. [36] evaluated the effectiveness of an IT decision support system in orthopedic treatment planning, which is built with the support of artificial intelligence. It allows the formulation of a personalized treatment plan.

Surgical simulators, image-guided navigation systems, and robotic assistive devices are improving current surgical practice and patient outcomes. New computer technologies enable the development of interactive patient-centered planners but also less invasive and more accurate interactive tools and sensors [37]. Qi et al. [38] studied the fixation of the proximal femoral anti-rotation intramedullary nail with the support of the computer-assisted navigation robot, compared to the classical one. The advantages highlight a precise minimally invasive operation performed in a shorter time, with less blood loss and transfusions, as well as reduced postoperative complications in the elderly. 

Levens et al. [39] investigated orthopedic patient compliance with patient-reported outcome measures. They concluded that reminders and interventions significantly improve response rates, which facilitates the advancement of medical care. In the absence of patient assessment in the medical office, e-mail reminders can be used, which increases the completeness of follow-up data [40].

With the support of these healthcare practices, we developed the content of the indicator IA1–Computerized clinical decision support systems. Its contents are shown in Table A5. This indicator is used to evaluate the basic medical activity of the hospital, located in the Implement (A) stage.

Orthopedic surgical specialty hospitals present the advantages of simplified elective surgery. But they are not equipped with all the facilities for the management of post-operative complications, hence the need for an inter-hospital transfer [41]. Transfer between facilities and hospital characteristics is associated with increased success in orthopedic interventions [42]. 

The quality of service provided by hospitals can be greatly improved by exploring and understanding the patient journey and transfer mechanisms. This requires the analysis of the space-time dynamics of transfer activities [43]. Along with understanding each patient’s journey, the quality of patient-centered care improves. Maas et al. [44] show that, in this process, digitization can facilitate placing patients’ wants and needs at the center of care. Improving transfer processes can be achieved by validating self-assessment and peer review of the quality of hospital transfers, which can be incorporated into certification programs.

Bradley et al. [45] show that inter-hospital transfer of major trauma patients contains deficiencies regarding adequate documentation of prehospital and early physiological data. Improving this situation can be achieved by professional medical organizations, which can also serve as powerful mediators of change. This requires increasing the visibility of transfers and mobilizing research funds. An interfacility transport program can function successfully if all the resources are in place to manage complex patients. One such resource is the use of extracorporeal membrane oxygenation [46]. 

With the support of these healthcare practices, we developed the content of the indicator IB1–Transfer evaluation mechanisms. Its contents are shown in Table A7. This indicator is used to evaluate the basic medical activity of the hospital, located in the Implement (B) stage.

#### 2.4.3. Indicators for Healthcare Facility Governance Evaluation

Local opinion leaders are health professionals named by their peers as “educational influencers” who influence the professional practice of peers [47]. The existence and recognition of local opinion leaders is a strategy for implementing improved medical behaviors of colleagues and all medical service providers. In different phases of the changes, they play a critical role in reducing the barriers to implementing behaviors [48]. 

Ayre et al. [49] show that the key activities that opinion leaders must carry out must be related to raising awareness and organizational commitment to the professionalization of innovative practices in orthopedics and to strategic and operational planning in orthopedic medical practice. Santos et al. [50] report an association between the existence of opinion leaders valued as professional champions and high instrumental use by the hospital. Recognizing their effectiveness requires the description of the targeted persons and the activities performed and the use of experimental study models to rigorously evaluate the effectiveness of the champions’ activities. 

The discussion panel between opinion leaders can highlight the potential for treatment and management of the prevalence of diseases such as osteoarthritis of the knee in young people. It is concluded that viscosupplementation with hyaluronic acid injections is a viable alternative [51]. Torrens et al. [52] are of the opinion that in shoulder surgery, opinion leaders from the orthopedic medical community are more influential compared to randomized controlled trials, even if they have results published in journals with a high impact factor.

With the support of these healthcare practices, we developed the content of the indicator EA1–Local opinion leaders’ existence and recognition. Its contents are shown in Table A9. This indicator is used to evaluate the basic medical activity of the hospital, located in the Evaluation (A) stage.

The construct of patient satisfaction is a complex process that depends on patients’ expectations before treatment and the nature and setting of treatment but also the context in which care takes place [53]. It should be assessed periodically on scales that have validity, reliability, and responsiveness as characteristics [54].

An important part of the patient-centered care model is the patient satisfaction evaluation mechanism. Murasko et al. [55] show that a popular measure used to evaluate orthopedic care is the patient satisfaction score. For this reason, it is necessary to regularly measure the satisfaction of patients but also of the medical staff and for this, there must be a dedicated procedure. Online evaluation of the medical service provided is a growing practice that affects the reputation of the orthopedic surgeon, directly or indirectly. But doctors who register a large number of reviews and a high rating are most likely to use reputation management strategies [56].

Yu et al. [57] show that, in general, orthopedic surgeons have a positive online image and the main factors that patients value when making an appointment with an orthopedic surgeon are promptness, professional knowledge, and friendliness. Unresolved patient complaints about surgeons are correlated with surgical complications and allegations of malpractice [58]. There must be designated persons to resolve complaints and also to measure patient satisfaction.

With the support of these healthcare practices, we developed the content of the indicator EB1–Monitoring mechanisms assignment. Its contents are shown in Table A11. This indicator is used to evaluate the basic medical activity of the hospital, located in the Plan Evaluation (B) stage.

#### 2.4.4. Indicators for Healthcare Facility Governance Continuous Improvement

Klockmann et al. [59] show that there is no comprehensive tool available to healthcare facilities for evaluating their organizational health literacy. They define such a tool that evaluates in five main categories: easy access and navigation; integration; quality management; communication with patients; and patient involvement. At the international level, the self-assessment framework of environmental hygiene in the health field has been validated, which helps healthcare facilities evaluate environmental hygiene and identify areas for improvement [60].

Patient self-reported outcome measures are useful in medical follow-up and scientific research. Lazrek et al. [61] conclude that the Walch-Duplay and Rowe scores, self-administered by questionnaire, are two assessment tools for shoulder instability that can be used clinically and reliably for patient follow-up. Symptomatic self-assessment can be conducted with the support of a web-based tool that helps patients and their caregivers take photos of painful areas and transmit some samples for remote diagnosis [62]. Early self-assessment of work-related musculoskeletal disorders using web-based applications can prevent severe symptoms and long-term consequences [63].

The clinical neuropsychological self-assessment policy of healthcare staff can be supported by audit tools. They facilitate the tracking, maintenance, and discussion of best medical practices over time [64].

With the support of these healthcare practices, we developed the content of the indicator RA1–Tools for self-assessment. Its contents are shown in Table A13. This indicator is used to evaluate the basic medical activity of the hospital, located in the Review (A) stage.

The complexity of healthcare makes service improvement a challenge, which can reduce clinical errors, streamline services, and make cost savings [65]. Teisberg et al. [66] opine that value in healthcare can be defined as the measured improvement in patient health outcomes relative to the cost of achieving that improvement. 

Integrated care is a means of substantially improving the outcomes of medical services. This is a key concern for regulatory authorities, decision makers, healthcare staff, and patients [67]. Systemic health system improvement requires a process of change in an adaptive context where personal and professional purpose is essential. Implementing systemic change must address the relationship between the vision, methods, and social dynamics of healthcare staff, patients, clients, and stakeholders.

In their turn, the leaders of healthcare facilities must produce initiatives to change medical services. Barson et al. [68] identified several initiatives regarding the modification of information confidentiality, the facilitation of professional training at the workplace, the use of service design methods in common with patients, and the use of data to improve the health status of the sickest and most vulnerable populations. Digital initiatives enable the use of telemedicine for continuous remote monitoring of critically ill patients [69]. An integrated vision of healthcare allows the use of means of communication in digital format for disease prevention, early detection and diagnosis, treatment, and healthcare [70].

With the support of these healthcare practices, we developed the content of the indicator RB1–Improvements in healthcare services. Its contents are shown in Table A15. This indicator is used to evaluate the basic medical activity of the hospital, located in the Review (B) stage.

### 2.5. Indicators Content and the Evaluation Model

In the continuation of the research, we designed the content of the indicators for the evaluation of the healthcare facility governance. For this purpose, we used the validated evidence for healthcare facilities governance presented in Section 2.4. The descriptions of the contents of the indicators are followed by the questions for their evaluation (see Table A1, Table A3, Table A5, Table A7, Table A9, Table A11, Table A13 and Table A15).

Each indicator has associated an importance variable described qualitatively and numerically in ascending order by irrelevant (0), unimportant (1), low importance (2), important (3), very important (4), and high importance (5).

The evaluation of the degree of fulfillment of the indicators is carried out on a 5-step scale (see Table A2, Table A4, Table A6, Table A8, Table A10, Table A12, Table A14 and Table A16). Each step is described textually and has associated values, as follows: not relevant (0), low (1), satisfactory (2), good (3), very good (4), and excellent (5).

The contents of the 8 indicators for the evaluation of the healthcare facility governance and the related evaluation grids are presented in detail in Table A1, Table A2, Table A3, Table A4, Table A5, Table A6, Table A7, Table A8, Table A9, Table A10, Table A11, Table A12, Table A13, Table A14, Table A15 and Table A16, as follows: Table A1. The indicator PA1–Decision-making structures and processes; Table A2. Scale for indicator PA1–Decision-making structures and processes; Table A3. The indicator PB1–Healthcare quality assurance processes design; Table A4. Scale for indicator PB1–Healthcare quality assurance processes design; Table A5. The indicator IA1–Computerized clinical decision support systems; Table A6. Scale for indicator IA1–Computerized clinical decision support systems; Table A7. The indicator IB1–Transfer evaluation mechanisms; Table A8. Scale for indicator IB1–Transfer evaluation mechanisms; Table A9. The indicator EA1–Local opinion leaders’ existence and recognition; Table A10. Scale for indicator EA1–Local opinion leaders’ existence and recognition; Table A11. The indicator EB1–Monitoring mechanisms assignment; Table A12. Scale for indicator EB1–Monitoring mechanisms assignment; Table A13. The indicator RA1–Tools for self-assessment; Table A14. Scale for indicator RA1–Tools for self-assessment; Table A15. The indicator RB1–Improvements in healthcare services; Table A16. Scale for indicator RB1–Improvements in healthcare services.

**Figure 4:**
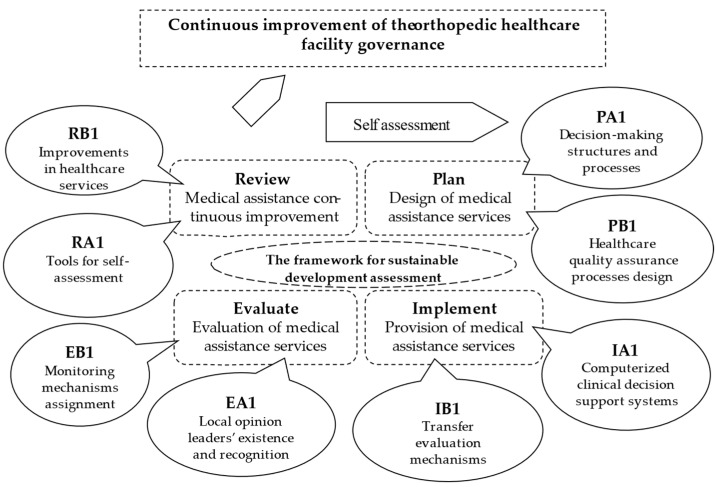
The healthcare facility governance continuous improvement cycle.

In the experimental part of the research, we validated in practice at a hospital with an orthopedic profile the content of the indicators designed for the evaluation of healthcare facility governance. For this purpose, after obtaining the approval of the ethics committee, we created a team of evaluators consisting of the head doctor of the department, the resident doctor, the chief assistant, and the person in charge of quality assurance. They audited the healthcare facility for a week in March 2024 and tracked the adequacy of the content of the indicators, the possibilities for improving the content of the indicators, and the evaluation grids but also the effectiveness of the evaluation through the proposed method. For this, we tested the 8 indicators in the sequence of the continuous improvement cycle (Figure 4) at the Emergency County Hospital Targu Mures (ECHTM), within the Orthopedics-Traumatology Department [71]. Corresponding to the first cycle planning stage, we evaluated the indicators PA1–Decision-making structures and processes and PB1–Healthcare quality assurance processes design. In the implementation phase, the indicators IA1–Computerized clinical decision support systems and IB1–Transfer evaluation mechanisms were used. The third phase continued with indicators EA1–Local opinion leaders’ existence and recognition and EB1–Monitoring mechanisms assignment. Finally, for the review, the indicators RA1–Tools for self-assessment and RB1–Improvements in healthcare services were employed.

## 3. Results

The eight designed indicators make up the indicator’s matrix associated with healthcare facility governance (Table 1).

The indicators matrix makes the connection between the stages of the quality cycle (column 1) and healthcare facility governance indicators (column 2). In the continuation of this section, our assessments regarding the indicators for healthcare facility governance at an emergency hospital in orthopedic profile are presented.

PA1–Decision-making structures and processes—ECHTM has defined the organizational structures involved in hospital accreditation. The responsibilities of the persons involved in accreditation from the medical services quality assurance department are defined in the organizational chart. The hospital obtained a low confidence grade at the ANMCS evaluation of 2021. This was due to an 85.59% degree of compliance with the health authorization of operation. The hospital has an accredited medical analysis laboratory in accordance with the ISO 15189 standard [72]. It also has a certified quality management system according to ISO 9001 [22].

PB1–Healthcare quality assurance processes design—The medical services provided are continuously improved through patient-centered consultations [73]. A procedure for implementing work protocols is applied, with the support of which medical services are constantly reviewed. There are 177 medical protocols formalized in the hospital’s departments that provide support for decisions regarding the clinical management of diseases.

IA1–Management of administrative data is conducted with the support of computer systems. The related logistics include fiber optic sections in a 600-m network, which offers back-up. There is an extensive park of computers, software licenses, multifunctional devices, and health card readers. Medical personnel benefit from this logistical support of computerized systems, which are used for clinical decisions, retrospective data analysis, preventive care, and disease management.

IB1–Transfer evaluation mechanisms—In 2023, 63,220 cases were presented to the UPU-SMURD emergency department from within the hospital, of which 11,289 cases were admitted and 10,207 cases were transferred. An evaluation of hospital transfers is carried out, which is ensured, depending on the severity, by car or air means. The transfer activity is not certified. Professional medical organizations are not involved in increasing the visibility of transfers. No research funds attracted with the support of professional medical organizations were identified.

EA1–Local opinion leaders’ existence and recognition—The medical staff in the department are professionally influenced by the local opinion leaders consisting of the chief physician and the quality management officer. They are also recognized through administrative mechanisms.

EB1–Monitoring mechanisms assignment—The measurement of patient satisfaction is carried out through a specific procedure of the healthcare facility, based on questionnaires accessible online at a web address on the hospital page, or by submitting suggestions in the patient’s box. The analysis of the results of the satisfaction surveys is published online. The satisfaction of the medical staff is also evaluated. The periodicity of evaluations is not strictly observed and the monitoring mechanisms have not been improved in the last year.

RA1–Tools for self-assessment—ECHTM is a public institution financed from the budget and from its own revenues, which has financial autonomy. Economic performance is evaluated using financial indicators. The hospital does not use self-assessment tools for environmental and social performance. The hospital has quantitative and qualitative self-assessment tools for patients, staff, and organizational health literacy, which require a series of improvements to the interface, integration, and patient involvement.

RB1–Improvements in healthcare services—The implementation of the rules and procedures of good medical practice and the follow-up of their revision, the implementation of therapies adapted to the objective needs of the patient, the parameters of the state of health, the pre-existing administrative and material conditions, and the follow-up of their changes and the real-time adaptation of the therapy to the changed conditions is in charge of the Medicines Commission within the hospital. At the request of the Clinical Department of General Surgery II, the Antibiotic Therapy Commission carried out the steps to introduce a new antimicrobial drug, Zolinef (Cefazolin), an antibiotic used for the treatment of infections, bone and joint infections, and skin and soft tissue infections, during or after surgery to prevent possible infections.

The values achieved for the indicators related to healthcare facility governance responsibility are registered in the self-assessment tool (Table 2).

**Figure 5:**
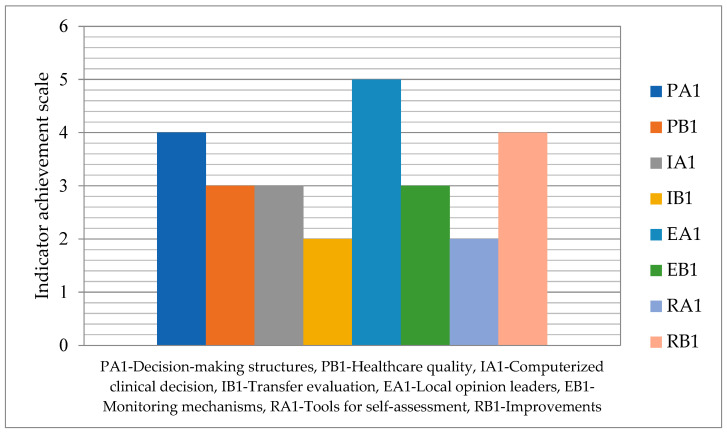
Achievement degree in healthcare facility governance responsibility.

The degree of achievement of indicators related to healthcare facility governance is depicted in Figure 5 on a scale in the range 1–5. In this domain, the indicators IB1–Transfer evaluation mechanisms and RA1–Tools for self-assessment have a minimum value of 2, while the highest value of 5 is recorded for the indicator EA1–Local opinion leaders’ existence and recognition.

**Figure 6:**
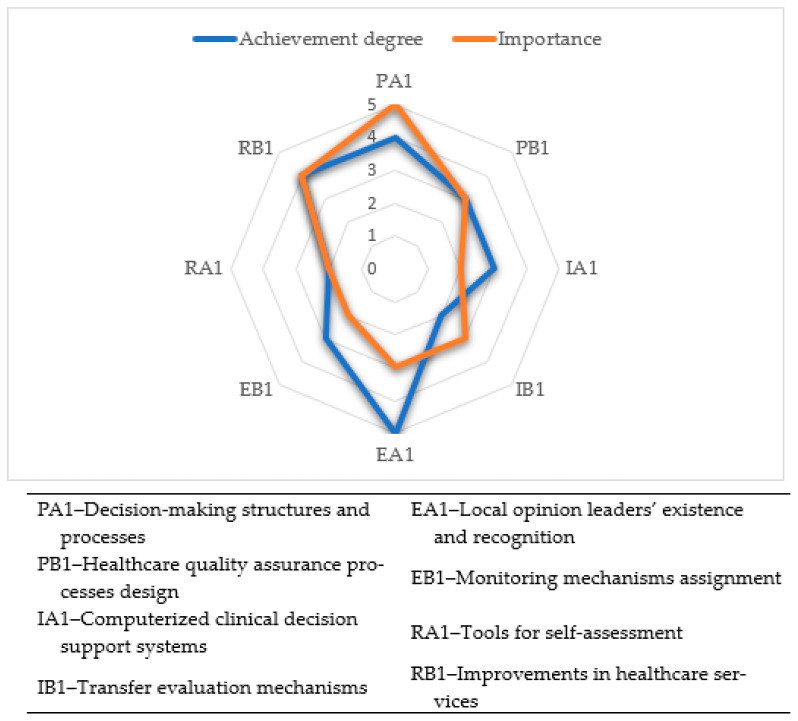
The healthcare facility governance evaluation graph.

A correlation between the importance and achievement degree of the indicators related to healthcare facility governance is depicted in the evaluation graph (Figure 6).

The sum of individual sustainability indicators from Table 2 reflects the global sustainability indicator for the healthcare facility governance (GS_HFG_):

(1) $${GS}_{HFG}=\sum_{i=1}^{8}S_{i}=\sum_{i=1}^{8}I_{i}\cdot A_{i}=82$$

The maximum value of global sustainability for the healthcare facility governance is the sum of the maximum values of the indicators that compose it (GSmax_HFG_)

(2) $${GSmax}_{HFG}=5\cdot \sum_{i=1}^{8}I_{i}=5\cdot 24=120$$

By reporting the percentage of the obtained values, the overall healthcare facility governance sustainability level is calculated (LGS_HFG_):

(3) $${LGS}_{HFG}=\frac{{GS}_{HFG}}{{GSmax}_{HFG\;}}\cdot 100=\frac{82}{120}\cdot 100=68.33\%$$

**Figure 7:**
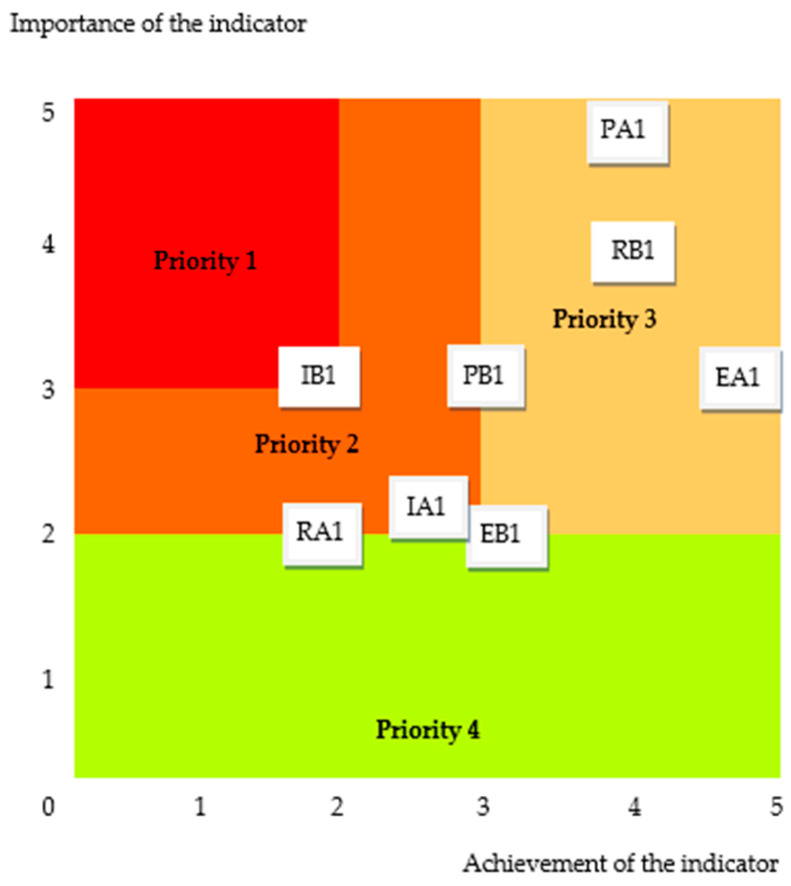
The assessment diagram for healthcare facility governance: PA1–Decision-making structures, PB1–Healthcare quality, IA1–Computerized clinical decision, IB1–Transfer evaluation, EA1–Local opinion leaders, EB1–Monitoring mechanisms, RA1–Tools for self-assessment, RB1–Improvements in healthcare services.

The extent to which the organizational governance requirements of the hospital are fulfilled are revealed by the result obtained. The Eisenhower matrix in Figure 7 indicates the priorities in the treatment of the 8 indicators. They were represented based on value couples.

For the current evaluation, to improve the healthcare facility governance, the highest priority should be given to the indicator IB1–Transfer evaluation mechanisms.

## 4. Discussion

The first conclusion of the practical testing was related to the content of the indicators, which was assessed as compatible with the national requirements for accreditation of healthcare facilities with beds [13] but also in outpatients [14], as well as with the international reference frameworks built for quality assessment in hospitals [15]. However, some contents of the indicators were also adjusted. Regarding the content of the developed methodology, the development of a glossary with terms specific to ensuring the quality and sustainability of medical processes was considered extremely useful. This would allow the correct understanding of the specific terminology and the correct appreciation of the references by the auditees and auditors. Also, depending on the profile of the hospital, the content of the indicators can be expanded with relevant medical practices from a wide spectrum of medical specialties. 

Another significant aspect revealed by our study is related to the human resources involved. The chief auditor must be a person with practical experience and a good planner and organizer but also a good connoisseur of the audited medical processes. He must impose himself through performance in the audited structures, which are usually preoccupied with current activities and tend to give limited importance to the audit. The audit program should be communicated in advance along with the audit evidence required to be prepared, so that the implementation is as efficient as possible. Easy communication channels must be established. Overall, we found that the implementation of the reference framework and the evaluation of the related indicators had a significant contribution to the increase in the responsibility of the health personnel regarding healthcare facility governance and organizational sustainability.

Following our study, we found that the indicator IB1–Transfer evaluation mechanisms, must be treated with priority to increase the degree of implementation of the organizational sustainability assurance system. This requires ECHTM to incorporate hospital transfers into its institutional certification program. It also needs to involve medical professional organizations more in increasing the visibility of transfers. At the same time, these organizations must contribute to the mobilization of research funds for hospital transfers. Contrary to the findings of Gualandi et al. [43], within the evaluated hospital, we observed that an analysis of the space–time dynamics of transfer activities is not carried out, which would allow a better understanding of the patient journey and transfer mechanisms, as well as improving the quality of the service offered.

In accordance with the study carried out by Shakibaei [25], we found that the hospital accreditation process contributed to positive changes in the management of the quality assurance processes of sanitary processes and to the increase in patient safety. Contrary to the conclusions of the study carried out by Mosadeghrad and Ghazanfari [27], we found that accreditation is not based on exhaustive models that also include sustainability aspects. This last aspect must be developed based on a model adopted by the hospital and is not required by law.

Like the study conducted by Goldchmit et al. [34], we found that the use of innovative patient training methods [74,75] has the effect of increasing the degree of patient-centered care simultaneously with the reduction in musculoskeletal pain. Contrary to the study carried out by Mills [35], we concluded that computerized support systems are not sufficiently well used to assist the process of diagnosing patients, monitoring anamnesis, clinical manifestations, paraclinical investigations, treatment, and rehabilitation. However, in agreement with the study carried out by El-Dawlatly et al. [36] we concluded that computer systems built with the support of artificial intelligence have the potential to support decisions in orthopedic treatment planning [76,77].

Regarding the measurement of patient and medical staff satisfaction, we found that the periodicity of evaluations is established but their implementation deviates from planning. Like the study conducted by Murasko et al. [55], the evaluation of orthopedic care is evaluated by the patient satisfaction score but the improvement measures formulated are not sufficiently visible to increase confidence in the evaluation system. In agreement with the findings of the study by de Klockmann et al. [59], we noted that quantitative and qualitative self-assessment instruments of patients, staff, and organizational health literacy require improved access, easier integration, and navigation so that they better support organizational governance. Consistent with the results reported by Barson et al. [68], our study revealed the need to develop an integrated vision of healthcare by encouraging the use of digital media in disease prevention, early detection and diagnosis, treatment, healthcare, and monitoring.

There are some limitations in the study conducted. We developed the indicators that allow the evaluation of healthcare facility governance based on the successful medical activities reported in the scientific literature. However, there may be some relevant results that are not yet reported and implicitly not included in the description of the indicators. Another limitation comes from the fact that we focused mainly on the specialty of orthopedics and an exhaustive exploration of other medical specialties would allow the content of the indicators to be enriched. We performed the validation in practice at a state emergency hospital but the ownership and organizational structures of the hospital can overcome this limitation of our study. Several future directions of study follow from these limiting considerations. First of all, the content of the indicators can be expanded to respond to as many medical specialties, organizational structures, and forms of hospital ownership as possible. The integration of the entire evaluation methodology in software would allow us, through digitization and with the support of IT tools, to simplify the evaluation effort and ensure its traceability.

## 5. Conclusions

In this research, we explored the ways of evaluating social responsibility regarding healthcare facility organizational governance as a component of sustainability. For this, we have developed a system made up of eight evaluation indicators that are part of a new evaluation framework for healthcare facilities. The construction of the indicators was based on successful medical practices reported by hospitals around the world. For each indicator, a qualitative and quantitative evaluation grid was designed on a scale from 0 to 5. The evaluation methodology assigns different importance to indicators on a qualitative and quantitative scale from 0 to 5. The practical validation of the content of the indicators at an orthopedic emergency hospital confirmed the adequacy of the result obtained with the support of the research methodology followed. The improvement measures are prioritized by evaluating the indicators in an Eisenhower matrix.

Through the practical evaluation, we have demonstrated the compatibility of the new reference framework and the indicators that make it up, with the quality assurance systems developed at the international level but also with the national legislation for accreditation in ambulatory care, as well as for sanitary units with beds. This allows its integration in relation to the other reference frames used by hospitals. The added value that a healthcare facility obtains by implementing this methodology consists of the application of sustainability requirements, organizational orientation, and its own staff, patients, and stakeholders toward sustainable development.

## Figures and Tables

**Table 1:** Healthcare facility governance indicator matrix of the H–S framework.

Quality Cycle	Healthcare Facility Governance Indicators
Plan Design of medical assistance services	PA1–Decision-making structures and processes
	PB1–Healthcare quality assurance processes design
Implement Provision of medical assistance services	IA1–Computerized clinical decision support systems
	IB1–Transfer evaluation mechanisms
Evaluate Evaluation of medical assistance services	EA1–Local opinion leaders’ existence and recognition
	EB1–Monitoring mechanisms assignment
Review Medical assistance continuous improvement	RA1–Tools for self-assessment
	RB1–Improvements in healthcare services

**Table 2:** Self-assessment tool for healthcare facility governance responsibility.

No.	Indicator Descriptive	Importance (Ii)	Achievement (Ai)	Sustainability Indicator (Si = Ii·Ai)
1	PA1–Decision-making structures and processes	5	4	20
2	PB1–Healthcare quality assurance processes design	3	3	9
3	IA1–Computerized clinical decision support systems	2	3	6
4	IB1–Transfer evaluation mechanisms	3	2	6
5	EA1–Local opinion leaders’ existence and recognition	3	5	15
6	EB1–Monitoring mechanisms assignment	2	3	6
7	RA1–Tools for self-assessment	2	2	4
8	RB1–Improvements in healthcare services	4	4	16

Ii—Importance, Ai—Achievement, Si—Sustainability Indicator.

## Data Availability

The data used in this study can be requested from the corresponding author.

## Appendix A

**Table A1:** The indicator PA1–Decision-making structures and processes.

Indicator	PA1–Decision-Making Structures and Processes
Description	Definition of organizational structures, responsibilities among staff and schemes/references used for accreditation containing scope and method of establishment, documentation, and implementation in relation to accreditation requirements (standards) and assessment tools.
Evaluation question	Are the organizational structures involved in the accreditation of the healthcare facility defined? Are the responsibilities of the people involved in accreditation defined? Are the normative requirements (accreditation/reference schemes) for accreditation identified? Is the scope of accreditation established? Are activities carried out to establish the quality management system according to the requirements (standards) identified and the assessment tools applied? Is the quality management system documented according to the identified requirements? Is the quality management system implemented according to the developed documentation?

**Table A2:** Scale for indicator PA1– Decision-making structures and processes.

Score [A]	Achievement	Content
0	Not relevant	–
1	Low	The organizational structures involved in the accreditation of the healthcare facility are defined and the responsibilities of the people involved in the accreditation are defined.
2	Satisfactory	Normative requirements, accreditation/referential schemes for accreditation are identified, and the scope of accreditation of the healthcare facility is established. The quality management system is documented.
3	Good	The healthcare facility is in the process of being accredited. The quality management system is implemented and assessment and monitoring tools are applied.
4	Very good	The healthcare facility is accredited with low confidence. The quality management system is implemented and is being certified.
5	Excellent	The healthcare facility is accredited with a high degree of trust. The quality management system is implemented and certified.

**Table A3:** The indicator PB1–Healthcare quality assurance processes design.

Indicator	PB1–Healthcare Quality Assurance Processes Design
Description	Patient-centered care seeks improvement: − Consultation processes; − The experiences of patients and care providers; − Knowing the patients; − Use of healthcare services; − Health and well-being.
Evaluation questions	Does the design of healthcare services consider patient-centered care? Are there improvements in services as a result of applying patient-centered care? Is patient consultation improved by focused care? Are patient experiences improved because of patient-centered care?

**Table A4:** Scale for indicator PB1–Healthcare quality assurance processes design.

Score [A]	Achievement	Content
0	Not relevant	–
1	Low	There are patient-centered care approaches but they are not input elements in the design of medical services.
2	Satisfactory	The approach in the healthcare facility is centered on the patient, by applying three basic conditions that create an atmosphere favorable to therapeutic change and growth: unconditional positive image, empathic understanding, and congruence. The design of medical services considers patient-centered care.
3	Good	The consultation is centered on the patient, through communication, setting priorities, and working in partnership to assess their circumstances. There are improvements in medical services because of the application of patient-centered care.
4	Very good	Patient-centered care creates a partnership relationship between the health staff and the patient and through the development and application of the consultation specific to general practice, effective doctor–patient relationships are obtained, respecting the principle of patient autonomy. Patient consultation is improved by applying patient-centered care.
5	Excellent	Patient-centered care develops the skills of continuous care, which emerge from the needs of the patient and achieves continuous and coordinated care. Patient experiences are improved as a result of patient-centered care.

**Table A5:** The indicator IA1–Computerized clinical decision support systems.

Indicator	IA1–Computerized Clinical Decision Support Systems
Description	The different functions in the system have at their disposal computerized support systems for clinical decisions, regarding − Diagnosis; − Reminders for preventive care or disease management; − Dosage and prescription of medicines.
Evaluation questions	Do medical staff have computerized support systems for clinical decisions? Are computerized support systems used to diagnose patients? Do computerized support systems provide reminders for preventive care or disease management? Are computerized support systems used for drug dosing and prescribing?

**Table A6:** Scale for indicator IA1–Computerized clinical decision support systems.

Score [A]	Achievement	Content
0	Not relevant	–
1	Low	Computer systems are used to manage administrative and/or financial data.
2	Satisfactory	Medical staff have at their disposal computerized clinical decision support systems that allow retrospective analysis of clinical data.
3	Good	Computerized support systems provide reminders for preventive care and disease management. Computerized support systems are used to diagnose patients.
4	Very good	Clinical decision support systems are used in assisting the process of establishing the diagnosis and forming the conclusion. Based on the data related to the patient’s condition, which is provided by the doctor, the system offers the best solution.
5	Excellent	Computerized support systems are used for: − Assisting the diagnostic process of patients; − Monitoring the anamnesis, clinical manifestations; paraclinical investigations, treatment; and rehabilitation; − Planning the resources needed to treat patients; − Training of medical specialists; − Prophylaxis.

**Table A7:** The indicator IB1–Transfer evaluation mechanisms.

Indicator	IB1–Transfer Evaluation Mechanisms
Description	The development and validation of self- and peer-review of the quality of hospital transfers is important and can be incorporated into certification programs. Medical professional organizations can also serve as powerful mediators of change, for example, by increasing the visibility of transfers and by mobilizing research funds.
Evaluation questions	Is the quality of hospital transfers assessed? Are professional medical organizations involved in transfer changes? It is evaluated, for example, with respect to increasing visibility and allocation of research funds.

**Table A8:** Scale for indicator IB1–Transfer evaluation mechanisms.

Score [A]	Achievement	Content
0	Not relevant	–
1	Low	The healthcare facility self-evaluates the quality of hospital-to-hospital transfers.
2	Satisfactory	A peer review of hospital transfers is carried out.
3	Good	Hospital transfers are incorporated into healthcare facility certification programs.
4	Very good	Medical professional organizations help increase the visibility of transfers.
5	Excellent	Medical professional organizations contribute to the mobilization of research funds for transfers.

**Table A9:** The indicator EA1–Local opinion leaders’ existence and recognition.

Indicator	EA1–Local Opinion Leaders’ Existence And Recognition
Description	The existence of local opinion leaders. Local opinion leaders are health professionals named by their peers as “educational influencers”.
Evaluation questions	Does the institutional culture have the recognition of local opinion leaders as a value? Are there local opinion leaders? Are they recognized within the community?

**Table A10:** Scale for indicator EA1–Local opinion leaders’ existence and recognition.

Score [A]	Achievement	Content
0	Not relevant	–
1	Low	The institutional culture values the recognition of local opinion leaders.
2	Satisfactory	Within the healthcare facility there are professionals who could become local opinion leaders.
3	Good	Within the healthcare facility there are professionals who are appreciated by the professional community as local opinion leaders.
4	Very good	Local opinion leaders are involved in the continuous improvement in medical services.
5	Excellent	Local opinion leaders are recognized within the community for their professionalism and contribution to improving medical services.

**Table A11:** The indicator EB1–Monitoring mechanisms assignment.

Indicator	EB1–Monitoring Mechanisms Assignment
Description	Designation of the responsible persons and the periodicity of carrying out the satisfaction evaluation.
Evaluation questions	Is there a specific procedure for measuring satisfaction? Are people/responsibilities assigned for measuring satisfaction? Is the periodicity of evaluations established?

**Table A12:** Scale for indicator EB1–Monitoring mechanisms assignment.

Score [A]	Achievement	Content
0	Not relevant	–
1	Low	Measuring the satisfaction of patients and medical staff is a concern of the healthcare facility.
2	Satisfactory	There is a specific healthcare facility procedure for measuring patient and medical staff satisfaction.
3	Good	People/responsibilities are assigned to measure satisfaction.
4	Very good	The periodicity of evaluations is established and they are carried out according to planning.
5	Excellent	Following the evaluations, conclusions, and suggestions for improvement, regarding the monitoring mechanisms are formulated.

**Table A13:** The indicator RA1–Tools for self-assessment.

Indicator	RA1–Tools for Self-Assessment
Description	Existing tools for qualitative and quantitative self-assessment of patients, staff, and organizational health literacy.
Evaluation questions	Are self-assessment tools used? What are the quantitative self-assessment tools? What are the qualitative self-assessment tools? What are the categories of assessments carried out?

**Table A14:** Scale for indicator RA1–Tools for self-assessment.

Score [A]	Achievement	Content
0	Not relevant	–
1	Low	The healthcare facility has tools for quantitative self-assessment of patients, staff, and organizational health literacy.
2	Satisfactory	The healthcare facility has tools for qualitative self-assessment of patients, staff, and organizational health literacy.
3	Good	Quantitative self-assessment tools of patients, staff, and organizational health literacy are used for periodic assessments.
4	Very good	Self-assessment tools are characterized by easy access and navigation, are integrated, and support quality management.
5	Excellent	Quantitative and qualitative self-assessment tools facilitate patient communication and engagement and are continuously improved.

**Table A15:** The indicator RB1–Improvements in healthcare services.

Indicator	RB1–Improvements in Healthcare Services
Description	Changes made based on suggestions from medical staff, patients, customers, and stakeholders.
Evaluation questions	What new medical services can be included in the healthcare facility’s portfolio? What is the development potential of the medical services offered?

**Table A16:** Scale for indicator RB1–Improvements in healthcare services.

Score [A]	Achievement	Content
0	Not relevant	–
1	Low	Medical staff, patients, clients, and interested parties formulate proposals to change medical services.
2	Satisfactory	Proposals to change medical services are analyzed by the management of the healthcare facility and proposals with potential for improvement are selected.
3	Good	New medical services are proposed that can be included in the healthcare facility’s portfolio.
4	Very good	There is potential for the development of the medical services offered in terms of the way of issuing the medical prescription and the way of granting the treatments.
5	Excellent	The use of digital means of communication is encouraged in the different phases of an integrated vision of healthcare: disease prevention, early detection and diagnosis, treatment, healthcare, and monitoring. Caregivers and healthcare professionals are trained and information is provided to patients.
